# Prevalence of asthma and associated factors among 6- and 7-year-old schoolchildren: a cross-sectional study in Santiago Island, Cabo Verde, 2022

**DOI:** 10.3389/fped.2026.1793771

**Published:** 2026-04-23

**Authors:** Maria do Céu Teixeira, Alexandra G. dos Santos, Isabel Inês Araújo, Maria Rosário O. Martins

**Affiliations:** 1 Faculdade de Ciências e Tecnologia, oNe HEalth ReSearch CenTer, Universidade de Cabo Verde, Praia, Cabo Verde; 2 Global Health and Tropical Medicine, GHTM, Associate Laboratory in Translation and Innovation Towards Global Health, LA-REAL, Instituto de Higiene e Medicina Tropical, IHMT, Universidade NOVA de Lisboa, UNL, Lisbon, Portugal

**Keywords:** asthma, Cape Verde, ISAAC, prevalence, schoolchildren, wheezing

## Abstract

Asthma and wheezing are important pediatric health problems in Africa, yet recent data from Portuguese-speaking countries remain scarce. The objective of this cross-sectional study is to estimate the prevalence of current asthma and associated factors among 6- and 7-year-old children on Santiago Island, Cabo Verde. From April to July 2022, 1,044 pupils from 43 randomly selected primary schools were surveyed using the validated ISAAC questionnaire and a 33-item risk-factor module completed by trained professionals based on caregiver reports, with current asthma defined as wheeze in the previous 12 months. The prevalence of current asthma was 10.5% (95% CI 8.7–12.4), while only 5% of children had a physician diagnosis; boys were more affected than girls (12.6% vs. 8.5%, *p* = 0.034), and the prevalence of rhinitis and eczema was 20.1% and 12.2%, occurring in 59% and 18% of asthmatic children, respectively. Complex sample logistic regression analysis indicates that current rhinitis [adjusted odds ratio (aOR): 7.86; 95% confidence interval (CI): 4.25–14.54] is a strongly associated comorbidity and antibiotic exposure in the first year of life (aOR: 2.10; 95% CI: 1.19–3.71) a risk factor for asthma development, whereas breastfeeding (aOR: 0.19; 95% CI: 0.06–0.55) is a protective factor. These findings indicate that about one in ten children has asthma in Santiago, with substantial under-diagnosis, and highlight the need for integrated management of allergic diseases, promotion of breastfeeding, rational antibiotic use, and strengthened primary care screening to reduce the burden of childhood asthma in Cabo Verde.

## 1 Introduction

Asthma is a chronic, heterogeneous inflammatory disease of the airways characterized by recurrent episodes of wheeze, cough, breathlessness and chest tightness that vary over time and in intensity (1). It affects an estimated 300 million people worldwide—about 14% of all children and 5%–10% of the total population—and its prevalence has been rising steadily over the last five decades (2, 3). Although mortality from asthma is relatively low, the disease contributes substantially to hospital admissions, school absenteeism and impaired quality of life, particularly in low- and middle-income countries where access to guideline-based diagnosis and treatment remains limited (4). Cross-national comparison of asthma trends has long been hindered by inconsistent case definitions and data-collection methods. The International Study of Asthma and Allergies in Childhood (ISAAC) addressed this limitation by developing a simple, validated questionnaire that has now been deployed in more than 100 countries; in fact, ISAAC provided the first truly standardized global snapshot of pediatric asthma in the 1990s (Phase I) and repeated the survey a decade later (Phase III). Those two waves documented a modest increase in the prevalence of *current asthma symptoms* among 6-7-year-olds—rising from roughly 11 % to 12 %—and, crucially, revealed marked geographic heterogeneity (Supplementary Table S1). Countries with low prevalences (≈ 2–4 %) were clustered mainly in parts of Asia, North Africa, Eastern Europe and the Eastern Mediterranean, whereas the highest prevalences (≈ 29–32 %) were observed in Southeast Asia, North America and Latin America (5–8). Building on the work of ISAAC, the Global Asthma Network (GAN) has extended the methodology to all ages. It has also recently reported that approximately one in ten children aged 6–7 and one in fifteen adults worldwide continue to experience wheezing. Furthermore, it found that around half of children with symptoms have severe disease (2, 5). A recent meta-analysis of 10 studies on asthma and/or wheeze prevalence in preschool and school-aged children in Africa, conducted between January 2012 and July 2023, suggested that asthma prevalence ranged from 1.70% to 20.85%. Four of the studies were conducted in South Africa and three in East Africa, with one each in Uganda, Tanzania and Ethiopia. One study was conducted in northern Africa (Morocco), and one in western Africa (Senegal). Finally, one study was conducted in Central Africa, specifically Angola (9).

The African continent is experiencing an epidemiological transition, which is defined as a shift from communicable to noncommunicable diseases. The prevalence of asthma and wheezing in African children has been associated with environmental exposures, such as household air pollution, which are common in low- and middle-income countries (10). However, there is a knowledge gap regarding asthma prevalence figures in core age groups in Cape Verde, a country characterized by rapid urban growth and exposure to Saharan dust (11), This is particularly true in Santiago, the nation's most populous island, which hosts 56% of the Cabo Verdean population. The ISAAC-aligned surveys were conducted over two decades ago on the islands of Sal and São Vicente, where the asthma prevalence among schoolchildren was estimated at 10.6% and 7.0%, respectively (12, 13). Additionally, a subsequent household study that included participants over six years of age reported a physician-diagnosed asthma prevalence of 6.2% across Santiago and São Vicente (14). Consequently, the true burden of asthma in this vulnerable group remains unknown, hindering the evidence-based planning of pediatric respiratory services.

The present investigation was motivated by evident gaps in pediatric asthma surveillance in Cabo Verde, coupled with the growing global burden of the disease. The study aimed to estimate the prevalence of asthma in 6- and 7-year-old schoolchildren on Santiago Island and to identify associated demographic, clinical, and environmental factors. Our study's findings aim to inform national public health strategies for the prevention, diagnosis, and control of asthma. The findings also provide a baseline for evaluating future interventions.

## 2 Methods

### 2.1 Study design and setting

We conducted a cross-sectional study between April and July 2022 among children aged 6–7 years enrolled in public basic-education schools on Santiago Island, Cabo Verde. There are 216 elementary schools on the island of Santiago, with 467 classes for 1st and 2nd graders. Data collection took place during the 2021/2022 academic year, under the COVID-19 preventive measures in place at that time, including masking, physical distancing and regular disinfection of equipment.

Cabo Verde, a lower-middle-income archipelago of ten volcanic islands in the eastern Atlantic, has experienced a notable epidemiological transition since gaining independence in 1975. Non-communicable diseases now surpass infectious illnesses in terms of morbidity and mortality (15).

At approximately 55 km by 35 km, Santiago is the country's largest island. It is administratively divided into nine municipalities, and in 2021, it was home to 56% of the national population, including 11,142 children in the target age group (11).

### 2.2 Participants and eligibility

All children aged six or seven years old who were enrolled in the first or second grade of basic education were eligible. To be included, children had to have lived with their parents or guardians for at least one year, and caregivers had to provide written informed consent. Those whose caregivers had not been responsible for them for at least one year were excluded.

### 2.3 Sample size and sampling procedure

The study adhered to the ISAAC protocol for population-based surveys. A list of all 216 basic education schools on Santiago Island was used as the sampling frame. To ensure proportional representation, 57 schools were selected through stratified random sampling based on municipality and school size (42 index schools and 15 reserves). All of the selected schools agreed to participate. Within each school, the required number of children were randomly selected from class lists provided by their teachers. Sample size was calculated using the single-proportion formula for prevalence studies. Er assume a prevalence (p) of 10.5% based on previous studies, a precision (d) of 2%, and a 95% confidence level. The minimum required sample size was set at 900 children. To strengthen inter-center comparability, as recommended by ISAAC, the sample size was increased to 1,044 participants, representing approximately 10% of all six- and seven-year-olds on the island.

### 2.4 Data-collection instruments and procedures

Data were collected using the validated Portuguese version of the ISAAC written questionnaire, which was adapted to the Cabo Verdean context and pre-tested for comprehension. The questionnaire included three ISAAC modules—Asthma (eight items), Rhinitis (six items), and Eczema (seven items)—plus a 33-item environmental and risk-factor module developed specifically for this study.

Interviewers administered the questionnaires to caregivers using tablet computers running REDCap® (Research Electronic Data Capture). This electronic data-collection platform ensured real-time consistency checks and secure storage. Before data collection began, the interviewers received structured training on the questionnaire content, field procedures, and ethical conduct.

### 2.5 Variables and operational definitions

**Current asthma (primary outcome)** is defined as a positive response to the question, “Has your child had wheezing in the past 12 months?”.

**Ever wheeze, exercise-induced wheeze, dry night-time cough, physician-diagnosed asthma, current rhinitis, current eczema** was used as defined by corresponding ISAAC items to describe children's health conditions (Supplementary Table S1).

**Independent variables:** sex, municipality (Praia, the capital, is considered urban and is compared with the interior, which is predominantly rural), breastfeeding, antibiotic use in the first year of life, paracetamol use, fast-food intake, physical activity, screen time, household fuel type, indoor cooling device, tobacco exposure, truck traffic, early-life and recent contact with cats/dogs, farm exposure, parental smoking and numerous socio-economic indicators (Supplementary Table S1).

### 2.6 Data management and statistical analysis

The data exported from REDCap was cleaned and analyzed using IBM SPSS® Statistics, version 31. Descriptive analyses were used to summarize the characteristics of the participants. Categorical variables were expressed as absolute and relative frequencies, while quantitative variables were expressed as means (standard deviation) or medians (interquartile range), depending on their distribution. We used Pearson chi-square or Fisher's exact test, as appropriate, to explore bivariate associations between qualitative variables. Complex sampling logistic regression was used to estimate crude and adjusted odds ratios, along with their respective 95% confidence intervals. Variables with *p* < 0.20 in the bivariate analysis were entered into a multivariable logistic regression model. To estimate robust standard errors, we used schools as clusters and a 5% significance level.

### 2.7 Bias and missing data

Several procedures were implemented to minimize bias. Selection bias was reduced by randomly sampling schools and achieving high participation across all sites, which were stratified by municipality. Information bias was minimized by using the validated ISAAC questionnaire, training interviewers to apply a standardized protocol, and conducting all interviews in the presence of the caregiver most familiar with the child's health. Data entry errors were avoided through real-time electronic data capture in REDCap with internal validation checks. All questionnaires were verified for completeness during fieldwork and again prior to data entry.

### 2.8 Ethical considerations

The study was conducted in accordance with the Declaration of Helsinki and Cabo Verdean legislation governing research involving human participants. Ethical approval was obtained from the National Health Research Ethics Committee of Cabo Verde (Process 2/2022), and authorizations were granted by the Ministry of Education and the participating school boards. Written informed consent was obtained from parents or guardians prior to participation, and confidentiality and anonymity were guaranteed throughout.

## 3 Results

**Figure 1:**
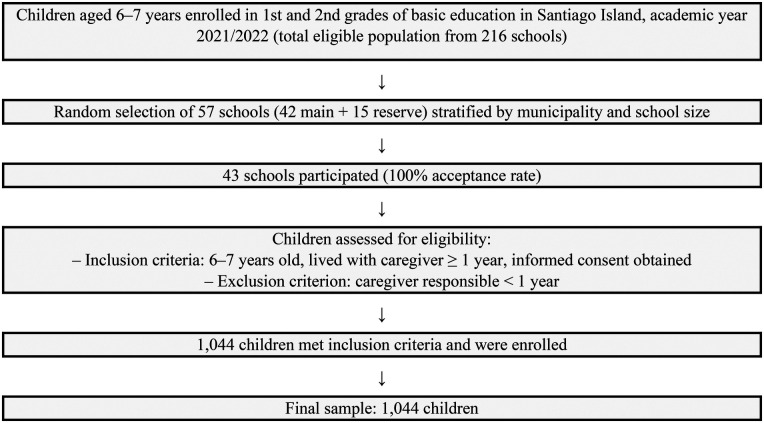
Flow diagram of participant selection and eligibility for the cross-sectional study of 6–7-year-old schoolchildren in Santiago island, cabo Verde (2022).

Figure 1 shows the process of school and participant selection, along with the inclusion and exclusion criteria (Flow diagram).

### 3.1 Participant characteristics

A total of 1,044 children, aged 6–7, from 43 public primary schools were included in the study. Girls represented 50.5% (*n* = 527) of the sample. Approximately 50% (*n* = 523) of the children lived in the urban municipality of Praia, and the remaining 50% lived in the predominantly rural interior. Most mothers/guardians (65.4%) had completed at least eight years of education (Table 1).

**Table 1:** Participants characteristics (*n* = 1,044).

Children and Parents characteristics	*n*	%
**Child Sex**		
Female	527	50.5
Male	517	49.5
**Place of residence**		
Praia municipality (urban)	523	50.1
Interior municipalities (predominantly rural)	521	49.9
**Mother/guardian's educational level**		
No schooling	28	2.7
1–7 years	333	31.9
8–12 years	488	46.7
>13 years	195	18.7
**Early life factors**		
Child took Paracetamol for fever in the first 12 months of life	673	64.5
Child took Antibiotics in the first 12 months of life	474	45.4
Child Breastfeeding	907	86.9

About one-third of the children lived with a cat during the first year of life, and 35 percent lived with a cat during the last year. About 43% lived with a dog during the first and last years. About 41% of mothers lived on a farm during pregnancy, and only 1.8% smoked (Table 2).

**Table 2:** Environmental characteristics (*n* = 1,044).

Environmental characteristics	n	%
Cat in first year of life	317	30.4
Cat in last 12 months	370	35.4
Dog in first year of life	449	43.0
Dog in last 12 months	459	43.9
Lived on a farm in first year of life	423	40.5
Lived on a farm during pregnancy	430	41.2
Mother smokes	18	1.7
Father smokes	104	10.0

### 3.2 Prevalence of asthma-related outcomes

Table 3 summarizes the estimated prevalence of asthma-related outcomes. Current asthma, defined as experiencing wheezing in the past 12 months, was estimated to affect 10.5% of the children (95%CI 8.7%;12.4%). We also estimated the prevalence of other related outcomes and comorbid conditions, such as current rhinitis and eczema. We also found that 5% of parents or guardians reported that their children had been diagnosed with asthma by a physician.

**Table 3:** Prevalence of asthma-related outcomes and co-morbid conditions in 6–7-year-olds (ISAAC definitions).

Outcome	n	%	Definition (ISAAC item)
Current asthma	110	10.5	Wheeze in the last 12 months
Ever wheeze	252	24.1	“Has your child ever had wheezing or whistling in the chest?”
Physician-diagnosed asthma	52	5.0	“Has a doctor ever told you…?”
Exercise-induced wheeze	84	8.1	Wheeze after physical activity, last 12 months
Dry night-time cough	315	30.2	Cough at night, last 12 months
Current rhinitis	210	20.1	Sneezing/runny or blocked nose without a cold, last 12 months
Current eczema	127	12.2	Itchy rash, last 12 months

### 3.3 Differences by sex and place of residence

As shown in Table 4, the prevalence of current asthma was higher in boys than in girls, at 12.6% and 8.5%, respectively (*p* = 0.034). Boys also had a higher prevalence of ever having wheezed than girls (27.7% vs. 20.7%, *p* = 0.008). The urban-rural difference in the prevalence of current asthma was modest and not significant, at 11.7% in Praia vs. 9.4% in interior municipalities (*p* = 0.235). Similar non-significant patterns were observed for other respiratory outcomes.

**Table 4:** Asthma-related outcomes by sex and place of residence.

Outcome	Female	Male	*p*-value*	Praia (urban)	Interior (rural)	*p*-value*
Ever wheeze	n (%)	n (%)		*n* (%)	*n* (%)	
Yes	109 (20.7)	143 (27.7)	0.008	131 (25.0)	121 (23.2)	0.491
No	418 (79.3)	374 (72.3)		392 (75.0)	400 (76.8)	
Current asthma (wheeze in last 12 months)	*n* = 527	*n* = 517		*n* = 523	*n* = 521	
Yes	45 (8.5)	65 (12.6)	0.034	61 (11.7)	49 (9.4)	0.235
No	482 (91.5)	452 (87.4)		462 (88.3)	472 (90.6)	
Physician-diagnosed asthma	*n* = 525	*n* = 517		*n* = 521	*n* = 521	
Yes	21 (4.0)	31 (6.0)	0.139	23 (4.4)	29 (5.6)	0.393
No	504 (96.0)	486 (94.0)		498 (95.0)	492 (94.4)	
Exercise-induced wheeze	*n* = 527	*n* = 515		*n* = 520	*n* = 522	
Yes	35 (6.6)	49 (9.5)	0.089	38 (7.3)	46 (8.8)	0.353
No	492 (93.4)	466 (90.5)		484 (92.7)	474 (91.2)	
Night-time cough (past 12 months)	*n* = 527	*n* = 516		*n* = 521	*n* = 522	
Yes	148 (28.1)	167 (32.4)	0.132	175 (32.6)	145 (27.8)	0.096
No	379 (71.9)	349 (67.6)		352 (67.4)		

* *p* value for Qui-Square or Fisher tests.

### 3.4 Co-morbid allergic disease

Of the 110 children with current asthma, 59.1% also had rhinitis, 18.2% had eczema, and 15.5% had all three conditions (asthma, rhinitis, and eczema). Only 25.5% had asthma alone (Table 5).

**Table 5:** Co-occurrence of current asthma with rhinitis and eczema among children with current asthma (*n* = 110).

Current asthma	Isolated asthma *n* (%)	Asthma and rhinitis *n* (%)	Asthma and eczema *n* (%)	Asthma, rhinitis and eczema *n* (%)
110	28 (25.5)	65 (59.1)	20 (18.2)	17 (15.5)

### 3.5 Factors associated with current asthma

Based on the literature review, the following variables were selected as possible factors associated with the presence or absence of current asthma:

- Sex
- Place of residence
- Comorbidities (rhinitis and eczema)
- Use of paracetamol for fever during the first 12 months of life
- Frequency of trucks passing on the street where the child lives
- Breastfeeding by the mother
- Presence of a cat or dog in the home during the first year of life
- Presence of a cat or dog in the home during the last 12 months
- Living on a farm during the first year of life
- Living on a farm during pregnancy
- Smoking by the mother or father

First, we estimated the crude odds ratio for all possible factors. The following variables with *p* < .20 were included as factors associated with the presence or absence of current asthma in the multivariable complex sampling logistic regression analysis: child's sex; current rhinitis; current eczema (lesions in flexural areas); breastfeeding; cat exposure in the first year; paracetamol use; and early antibiotic use. The crude odds ratios are presented in Supplementary Table S4, and the adjusted odds ratios and their respective 95% confidence intervals (CIs) are shown in Table 6.

**Table 6:** Factors associated with current asthma in 6–7-year-olds children, Santiago island, Cape Verde (Complex sampling logistic regression results).

Variables	Adjusted Odds Ratio	*p* value	95% CI	
Sex (male vs. female)	0.82	0.56	0.414	1.632
Current rhinitis (Yes vs. No)	7.862	<.001	4.251	14.54
Breastfeeding (Yes vs. No)	0.186	0.003	0.063	0.548
Current Eczema (Yes vs. No)	0.876	0.748	0.382	2.008
Antibiotics in the first year of life (Yes vs. No)	2.102	0.012	1.189	3.714
Cat in first year of life (Yes vs. No)	0.589	0.066	0.334	1.036
Intercept	0.323	0.007		

We identified two comorbidities and one risk factors that increase the odds of having current asthma: current rhinitis [adjusted odds ratio (aOR) = 7.87], current eczema (aOR = 0.88) and early-life antibiotic exposure (aOR = 2.1), However, the the odds ratio for current eczema was not significantly different from one. Our results also suggested three protective factors: breastfeeding (aOR = 0.19), having a cat in the first year of life (aOR = 0.59), and being a girl (aOR = 0.82). However, the latter two odds ratio were not significantly different from one at the 5% significance level.

## Discussion

This study provides the first population-based estimate of asthma prevalence among six- and seven-year-old children on Santiago Island in Cape Verde. We estimate the prevalence of current asthma to be 10.5%. Our results suggest that asthma affects boys more than girls, and half of the symptomatic children had not received a medical diagnosis. Most asthmatic children had concurrent rhinitis, highlighting the close clinical overlap between the two conditions. Current rhinitis was identified as a strongly associated comorbidity and antibiotic use in early life as a risk factor that increase the odds of having current asthma. Conversely, breastfeeding was identified as a protective factor.

The findings revealed that asthma is a significant public health issue for this age group in Santiago. The estimated prevalence of 10.5% is comparable to the 10.6% figure reported in Sal Island and higher than the 7.0% figure found in São Vicente over two decades ago (9.18). It is also higher than the 6.2% prevalence of physician-diagnosed cases reported in a household survey that included all age groups in Santiago and São Vicente Islands (14). Taken together, these results suggest that asthma remains a significant health burden, with underdiagnosis still common. Only around half of children with symptoms are ever medically identified as asthmatic. This may be due to limited access to spirometry and pediatric specialists, caregiver stigma surrounding inhaled therapy, and diagnostic uncertainty in preschoolers.

The higher prevalence in boys (12.6% vs. 8.5%) mirrors the sex pattern observed in most prepubertal groups and may be due to smaller airways in boys or sex-specific immune modulation (16). The modest and insignificant urban-rural differences suggest that risk factors are distributed relatively evenly across Santiago Island, where urbanization has spread to many formerly rural municipalities (11). Conversely, the strong co-occurrence of asthma and rhinitis (59%) is consistent with the “one airway” paradigm and the shared pathophysiology observed in ARIA and GAN (17). The prevalence of rhinitis and asthma underscores the importance of incorporating allergic disease screening and management into pediatric primary care.

The risk and protective factors identified in our study align with the findings of the ISAAC Phase III and contemporary meta-analyses (9, 18, 19). Early antibiotic use may disrupt the gut and airway microbiota, which can skew immune maturation towards a Th2 phenotype. In contrast, the protective effect of breastfeeding is biologically plausible, considering the immunological and microbiological benefits of breast milk. These benefits include passive immunity, regulation of gut flora, and a reduction in respiratory infections during infancy (20). The nearly significant association with exposure to infant cats supports the hygiene/endotoxin hypothesis that early microbial diversity can downregulate atopic responses. However, the study lacked sufficient power to confirm this pattern.

Overall, our study has several strengths. First, we used a large, randomly selected sample covering half of the national target population and we estimate a logistic regression using a complex sampling approach, with schools taken as clusters. Second, we used the validated ISAAC instrument, which enables global comparability. Finally, we used electronic data capture with built-in logic checks. However, the study also has some limitations. The cross-sectional design precludes causal inference, recall bias and limited caregiver literacy may have affected the accuracy of the questionnaires. Furthermore, unmeasured confounders, such as viral wheezing phenotypes and allergen sensitization, were not captured. Future work would benefit from objective lung function or IgE measurements and longitudinal follow-up. Despite measures taken to minimize bias, residual confounding factors and caregiver recall errors may have influenced some of the observed associations.

Due to the study's design and high participation rate, the results are representative of 6- to 7-year-old children in Santiago Island. By extension, the results are likely applicable to other urbanizing contexts within Cabo Verde. However, the results may not apply to older age groups or smaller islands with different environmental and socioeconomic characteristics.

### 4.1 Public health and clinical implications

The findings suggest that there is a substantial, yet largely undiagnosed, burden of childhood asthma in Cabo Verde. The observed associations emphasize the need to improve the early detection and management of asthma and allergic diseases in primary care. A policy priority should be establishing a National Asthma Control Program that includes training for primary care workers, access to affordable inhaled corticosteroids, and public education to reduce stigma surrounding treatment. Promoting exclusive breastfeeding and ensuring the judicious use of antibiotics in infancy may reduce the risk of asthma. To elucidate causal pathways and geographic heterogeneity, cohort studies incorporating lung function testing, allergen sensitization profiles, and environmental monitoring across multiple islands are necessary. Additionally, prevalence studies of adolescents and adults would provide a comprehensive picture of the life course and inform integrated strategies for chronic respiratory diseases.

## 5 Conclusion

This study provides the first island-wide, population-based estimate of asthma symptoms among six- and seven-year-old children in Santiago, Cabo Verde. Of these children, one in ten experienced wheezing in the past year, and only half of them had ever received a medical diagnosis, highlighting a significant under recognition of asthma that contributes to avoidable morbidity. Consistent with the “one-airway” concept, current rhinitis was the strongest predictor of asthma. Early-life antibiotic use was identified as a modifiable risk factor, and breastfeeding was identified as a protective factor. Taken together, these findings emphasize the need for systematic case finding and guideline-based management in primary care, integration of rhinitis screening into asthma programs, strengthened antimicrobial stewardship, and promotion of breastfeeding. Additionally, there is a need for investment in longitudinal research to track the evolving epidemiology of atopic disease across the archipelago. Implementing a national asthma control program that focuses on training, inhaled therapy availability, and public education should now be prioritized to reduce the burden of childhood asthma in Cabo Verde.

## Data Availability

The raw data supporting the conclusions of this article will be made available by the authors, without undue reservation.
