# Supplementary material for: Prevalence of asthma and associated factors among 6- and 7-year-old schoolchildren: a cross-sectional study in Santiago Island, Cabo Verde, 2022
Source: Front Pediatr. 2026 Apr 23;14:1793771. doi: 10.3389/fped.2026.1793771 (PMC13149253; doi:10.3389/fped.2026.1793771)
Supplement: Supplementary file 1 [file Table1.docx]

Supplementary Tables

Supplementary Table S1. ISAAC Questions used in our study

| Variable group | Analysis variable (short name) | ISAAC / risk-factor question (English translation) | Original response options | Coding used for analysis* |
| --- | --- | --- | --- | --- |
| Primary outcome | Current asthma (current_wheeze_12m) | “Has your child had wheezing or whistling in the chest **in the past 12 months**?” | Yes / No | Yes = 1, No = 0 |
|  | Ever wheeze (ever_wheeze) | “Has your child **ever** had wheezing or whistling in the chest?” | Yes / No | Yes = 1, No = 0 |
|  | Exercise-induced wheeze (ex_wheeze) | “In the past 12 months, has your child’s chest sounded wheezy during or after exercise?” | Yes / No | Yes = 1, No = 0 |
|  | Night-time cough (night_cough) | “In the past 12 months, has your child had a dry cough at night when they did **not** have a cold?” | Yes / No | Yes = 1, No = 0 |
|  | Physician-diagnosed asthma (md_asthma) | “Has a doctor **ever** told you that your child has asthma?” | Yes / No | Yes = 1, No = 0 |
| Co-morbid allergy | Current rhinitis (rhinitis_12m) | “In the past 12 months, has your child had sneezing or a runny/blocked nose when they **did not** have a cold?” | Yes / No | Yes = 1, No = 0 |
|  | Current eczema (eczema_12m) | “In the past 12 months, has your child had an itchy rash that came and went for at least six months?” | Yes / No | Yes = 1, No = 0 |
| Demographics | Sex (sex) | School record (“Male” / “Female”) | Male / Female | Male = 1, Female = 0 |
|  | Urbanicity (urban) | Municipality of the school (Praia = urban; all others = rural) | Praia / Other | Urban = 1, Rural = 0 |
| Early-life exposures | Ever breast-fed (bf_ever) | “Was your child **ever** breast-fed?” | Yes / No | Yes = 1, No = 0 |
|  | Antibiotics 1st year (abx1y) | “Did your child receive antibiotics during the **first year of life**?” | Yes / No / Don’t know | Yes = 1, No/ DK = 0 |
|  | Paracetamol 1st year (pcm1y) | “Did your child receive paracetamol during the **first year of life**?” | Yes / No / Don’t know | Yes = 1, No/ DK = 0 |
|  | Cat exposure 1st year (cat1y) | “Was there a cat living in the home during your child’s **first year of life**?” | Yes / No | Yes = 1, No = 0 |
|  | Dog exposure 1st year (dog1y) | Same wording for dogs | Yes / No | Yes = 1, No = 0 |
| Current environmental factors | Solid-fuel cooking (solid_fuel) | “What is the main cooking fuel in your household?” | Wood / Charcoal / Gas / Electric | Wood/Charcoal = 1, Clean = 0 |
|  | Passive smoking (passive_smoke) | “Does anyone smoke **inside** your home?” | Yes / No | Yes = 1, No = 0 |
|  | Heavy truck traffic (truck_fx) | “How often do heavy trucks pass in the street where you live?” (Never / Occasionally / Frequently) | 3-level categorical | Frequent (≥ 1 day wk⁻¹) = 1, Else = 0 |
| Lifestyle factors | Fast-food ≥ 1 × week (fast_food) | “Does your child eat fast-food at least once a week?” | Yes / No | Yes = 1, No = 0 |
|  | Screen time ≥ 2 h day (screen2h) | “On a typical day, how many hours does your child spend on screens (TV, computer, phone, tablet)?” (< 2 h / 2–4 h / > 4 h) | 3-level categorical | ≥ 2 h = 1, < 2 h = 0 |

* All binary variables coded as 1 = present, 0 = absent.

Supplementary Table S2. Crude odds ratio

| Variables | n | No asthma | Asthma | *p-value* | Crude Odds Ratio | (CI95%) |
| --- | --- | --- | --- | --- | --- | --- |
| Child Sex | | n (%) | n (%) |  |  |  |
| Female | 527 | 482 (91,5) | 45 (8,5) | 0,035 | 0,649 | 0,435 - 0,970 |
| Male | 517 | 452 (87,4) | 65 (12,6) |  |  |  |
| Residence | | | | |  |  |
| Praia (urban) | 523 | 462 (88,3) | 61 (11,7) | 0,235 | 1,272 | 0,855 - 1,892 |
| Interior (rural) | 521 | 472 (90,6) | 49 (9,4) |  |  |  |
| Current rhinitis | | | | |  |  |
| No | 834 | 789 (94,6) | 45 (5,4) | <0,001 | 7,86 | 5,168 -11,954 |
| Yes | 210 | 145 (69,0) | 65 (31,0) |  |  |  |
| Current Eczema | | | | |  |  |
| No | 917 | 827 (90,2) | 90 (9,8) | 0,041 | 1,718 | 1,016 -2,903 |
| Yes | 127 | 107 (84,3) | 20 (15,7) |  |  |  |
| Paracetamol for fever in the first 12 months of life | | | | |  |  |
| No | 330 | 303 (91,8) | 27 (8,2) | 0,074 | 1,514 | 0,958 -2,393 |
| Yes | 673 | 593 (88,1) | 80 (11,9) |  |  |  |
| Antibiotics in the first 12 months of life | | | | |  |  |
| No | 472 | 438(92,8) | 34 (7,2) | <0,001 | 2,195 | 1,424 - 3,382 |
| Yes | 474 | 405 (85,4) | 69 (14,6) |  |  |  |
| Breastfeeding | | | | |  |  |
| No | 28 | 20 (71,4) | 8(28,6) | 0,006 | 0,281 | 0,121 - 0,655 |
| Yes | 907 | 767 (89,9) | 102 (10,1) |  |  |  |
| Cat in first year of life | | | | |  |  |
| No | 724 | 637 (88,0) | 87 (12,0) | 0,021 | 0,573 | 0,354 - 0,926 |
| Yes | 317 | 294 (92,7) | 23 (7,3) |  |  |  |
| Cat in last 12 months | | | | |  |  |
| No | 672 | 602(89,6) | 70 (10,4) | 0,843 | 1,042 | 0,691 - 1,572 |
| Yes | 370 | 330 (89,2) | 40 (10,8) |  |  |  |
| Dog in first year of life | | | | |  |  |
| No | 594 | 533 (89,7) | 61 (10,3) | 0,738 | 1,07 | 0,719 - 1,593 |
| Yes | 449 | 400 (89,1) | 49 (10,9) |  |  |  |
| Dog in last 12 months | | | | |  |  |
| No | 582 | 521 (89,5) | 61 (10,5) | 0,919 | 1,021 | 0,686 - 1,519 |
| Yes | 459 | 410 (89,3) | 49 (10,7) |  |  |  |
| Lived on a farm in first year of life | | | | |  |  |
| No | 618 | 552 (60,1) | 64 (56,6) | 0,898 | 1,027 | 0,686 - 1,537 |
| Yes | 423 | 378 (89,4) | 45 (10,6) |  |  |  |
| Lived on a farm during pregnancy | | | | |  |  |
| No | 612 | 552 (90,2) | 65 (11,6) | 0,346 | 1,211 | 0,814 - 1,801 |
| Yes | 430 | 380 (88,4) | 106 (42,2) |  |  |  |
| Mother smokes | | | | |  |  |
| No | 1025 | 916 (89,4) | 109 (10,6) | 0,711 | 0,494 | 0,065 - 3,751 |
| Yes | 18 | 17 (94,4) | 1(5,6) |  |  |  |
| Father smokes | | | | |  |  |
| No | 940 | 839 (89,3) | 101 (10,7) | 0,633 | 0,84 | 0,411 - 1,719 |
| Yes | 95 | 86 (0,90) | 9 (0,10) |  |  |  |
